# Transcriptomic analysis of plasma-derived small extracellular vesicles reveals the pathological characteristics of normal tension glaucoma

**DOI:** 10.20517/evcna.2024.45

**Published:** 2024-08-19

**Authors:** Sheng-Lan Xu, Jun-Hua Li, Wen-Meng Zhang, Meng-Jun Fu, Hui-Min Xing, Hua Ma, Xian-Hui Gong, Rong-Han Wu, Yuan-Bo Liang, Ren-Zhe Cui, Zai-Long Chi

**Affiliations:** ^1^State Key Laboratory of Ophthalmology, Optometry and Visual Science, Eye Hospital, Wenzhou Medical University, Wenzhou 325027, Zhejiang, China.; ^2^National Clinical Research Center for Ocular Diseases, Eye Hospital, Wenzhou Medical University, Wenzhou 325027, Zhejiang, China.; ^3^Department of Ophthalmology, Affiliated Hospital of Yanbian University, Yanji 133001, Jilin, China.; ^#^Authors contributed equally.

**Keywords:** Normal tension glaucoma, small extracellular vesicles, mitochondrial dysfunction, biomarker, let-7b-5p

## Abstract

**Aim:**

Normal tension glaucoma (NTG) is a common optic neuropathy that can be challenging to diagnose due to the intraocular pressure remaining within the normal range. Early diagnosis and intervention are crucial for the effective lifelong management of patients.

**Methods:**

This study recruited a total of 225 participants. Small extracellular vesicles (sEVs) RNA from circulating plasma was analyzed via transcriptomic sequencing, and its expression levels were verified by quantitative real-time polymerase chain reaction (qRT-PCR). Logistic regression, linear regression, and receiver operating characteristic (ROC) curve analyses were performed to examine the association of biomarkers with clinicopathological characteristics.

**Results:**

Analysis of sEVs mRNAs in NTG patients revealed mitochondrial dysfunction and enrichment of central nervous system degenerative pathways, reflecting the pathological features of NTG. Compared with those in the controls, the expression levels of sEVs let-7b-5p in the plasma of NTG patients were significantly lower, with an area under the curve (AUC) of 0.870 (95%CI: 0.797-0.943) (*P* < 0.0001), and the AUC combined with age was 0.923 (95%CI: 0.851-0.996) (*P* < 0.0001). In addition, we found that let-7b-5p levels were significantly correlated with the severity and visual field defects of NTG patients and had good specificity compared with other ophthalmic diseases.

**Conclusion:**

The sEVs RNA signatures in circulating plasma from NTG revealed mitochondrial dysfunction and that sEVs let-7b-5p can be a useful noninvasive biomarker for NTG.

## INTRODUCTION

Glaucoma is caused by retinal ganglion cell (RGC) degeneration, leading to progressive impairment of the visual field^[1]^. Normal tension glaucoma (NTG) is known to be more prevalent than the high-pressure form of primary open-angle glaucoma (POAG) in Asian populations, with approximately 70% of all POAG cases in Chinese populations also manifesting as NTG^[2-4]^. NTG is characterized by a lack of obvious symptoms, often leading to late-stage diagnoses. Patients typically have already developed serious visual field defects and visual impairments, which significantly jeopardize their visual function. Therefore, the early detection and diagnosis of NTG are pressing issues that need to be addressed.

The mechanism of NTG is complex and is not completely understood^[5]^. Numerous studies have reported that the translaminar pressure gradient^[6]^, mitochondrial dysfunction^[7]^, and oxidative stress are involved in the pathogenesis of NTG^[8,9]^. In addition, increasing evidence also supports the notion of a link between neurodegeneration in the brain and the optic nerve^[10-12]^. Interestingly, mitochondrial dysfunction is a known common feature of central nervous degenerative diseases^[13]^. The inability to obtain fresh pathological retinal tissues is one of the bottlenecks restricting research into the mechanism of disease. Early diagnosis and early intervention are important for glaucoma management, preventing impairment of visual function as much as possible throughout life. Lowering intraocular pressure (IOP) is the only recognized treatment^[6]^, but there are still some patients with progressive visual impairment and even blindness. Visual field examination and optical coherence tomography (OCT) analysis are the most commonly used early diagnostic methods in clinical practice, but are insufficient. In fact, up to half of the RGCs are already degenerated when diagnosed in patients with both POAG and NTG because there are no obvious symptoms during the early stages^[14]^. Therefore, there is a need to develop more precise methods, such as molecular biomarkers, for early diagnosis and monitoring of disease progression.

Recent developments have led to renewed interest in small extracellular vesicles (sEVs)^[15]^ as biomarkers for various diseases, including neurodegenerative diseases, and they are more reliable than conventional specimens^[16-18]^. sEVs can cross the blood-brain barrier and have low immunogenicity, thereby receiving much attention as potential biomarkers and drug-delivery tools for neurodegenerative diseases^[18-21]^. Based on the lipid bilayer membrane structure of sEVs, which encapsulate coding or noncoding RNAs, they can avoid degradation, and studies have also shown that miRNAs are prominently enriched in the sEVs RNA distribution^[22]^.

sEVs play a crucial role in intercellular communication by transporting various molecules to recipient cells. These cargo molecules can modulate signaling mechanisms and downstream gene expression in recipient cells, thereby contributing to the regulation of various biological processes. The mechanism of sEVs-mediated modulation of signaling and gene expression provides a comprehensive understanding of how sEVs participate in the regulation of various biological processes^[23-25]^.

sEVs are secreted by live cells and released into extracellular spaces and body fluids. Studies have shown that, unlike secreted molecules, sEVs can be taken up by other cells and then released after intracellular reactions^[26]^. Therefore, the sEVs in the circulating blood may represent the products after the reaction but also reveal systemic regulatory mechanisms in the body. These characteristics make them potential sources of biomarkers and hidden pathological information regarding complex diseases. An increasing number of diagnostic biomarkers of sEVs from body fluids have been identified for different diseases, providing an opportunity for accurate diagnosis and evaluation of disease progression in patients^[27-29]^. Notably, combinations of three mRNA expression levels of urine exosomes to predict high-grade prostate cancer and the related test kits have been approved by the Food and Drug Administration (FDA) for clinical application^[30,31]^. However, no studies on the exosome-based identification of noninvasive body fluid biomarkers for ocular neurodegenerative diseases have been reported.

As surgical treatment is not administered to patients with NTG, corresponding intraocular fluid samples cannot be procured^[32]^. In this study, we isolated circulating plasma sEVs from NTG patients, and the RNA signatures were explored by high-throughput sequencing. Then, possible biomarker identification and further verification were conducted via quantitative polymerase chain reaction (qPCR) and correlation analyses. The results confirmed that mitochondrial coding genes were significantly upregulated in NTG patients. Moreover, we found that sEVs let-7b-5p levels were correlated with visual field defects in NTG patients. The sEVs let-7b-5p levels may be a possible biomarker for the clinical diagnosis or evaluation of the disease progression of NTG.

## METHODS

### Participants

The criteria for the diagnosis of NTG included the following: (1) a maximum IOP not exceeding 21 mmHg; (2) the absence of abnormal anterior chamber angles; (3) the characteristic optic disc damage indicative of glaucoma (cup-to-disc ratio > 0.6); (4) corresponding visual field defects; and (5) the absence of other retinal diseases, ocular trauma, or systemic conditions. Demographic data including name, sex, age, history of ocular disease, and systemic clinical symptoms were recorded for all participants. Both eyes or the affected eyes underwent slit lamp examination, gonioscopy, IOP measurement, fundus imaging, OCT, and visual field (VF) testing. The exclusion criteria included systemic hemorrhagic, coagulation, and immune disorders; severe cardiovascular diseases; heart or renal failure; metabolic disorders such as hypertension and diabetes; other intraocular neovascular diseases; and refractive media opacity precluding clear fundus visualization.

The inclusion criteria for the control group consisted of patients diagnosed with age-related cataracts or ametropia who met the following criteria: (1) the absence of abnormal anterior chamber angles; (2) no signs of glaucomatous optic disc damage; (3) no corresponding visual field defects; and (4) the absence of other retinal diseases, ocular trauma, or systemic illnesses. The exclusion criteria included glaucoma and other retinal diseases; systemic hemorrhagic, coagulation, and immune disorders; severe cardiovascular diseases; heart or renal failure; metabolic disorders such as hypertension and diabetes; other intraocular neovascular diseases; and refractive media opacity impairing fundus visualization.

Written informed consent was obtained from all participants who did not receive any compensation or incentives for their involvement. The study was conducted in accordance with the Declaration of Helsinki and approved by the Ethics Committee of the Eye Hospital Affiliated with Wenzhou Medical University (2022-091-K-69). The study was based on the relevant provisions of the Regulations on the Administration of Human Genetic Resources of the People’s Republic of China, and had passed the review of the Office of the Administration of Human Genetic Resources of China.

### sEVs isolation

Peripheral blood was collected from the patients using tubes containing ethylenediaminetetraacetic acid (EDTA). Blood samples were centrifuged at 3,000 × *g* for 10 min. The supernatant plasma was recovered and stored at -80 °C until use. The samples were used for sequencing and validation experiments within one year. EVs were isolated from the plasma using differential centrifugation combined with the total sEVs precipitation method. The plasma was centrifuged at 2,000 × *g* for 20 min; then, the supernatant was transferred into a new tube and centrifuged at 10,000 × *g* for 20 min. Then, one-third of the supernatant volume of precipitation reagent (C10110-2, RiboBio, Guangzhou, China) was added and incubated at 4 °C for 20 min; the suspension liquid was centrifuged at 3,000 × *g* for 15 min; finally, sEV pellets were resuspended in phosphate-buffered saline (PBS). The morphology and size of the EVs were identified using transmission electron microscopy (TEM), nanoparticle tracking analysis (NTA), and western blotting.

### Western blot

Western blotting was performed to detect sEVs markers, including Alix, TSG101, and Syntenin proteins. Briefly, proteins extracted from sEVs were separated by 10% sodium dodecyl sulfate-polyacrylamide gel electrophoresis (SDS-PAGE), and gels were transferred to polyvinylidene fluoride (PVDF) membranes (Bio-Rad, USA). Membranes were blocked in 5% skimmed milk (Beyotime, China) for 2 h and incubated with primary antibodies, including anti-Alix (1:500, rabbit monoclonal, PTM-6407, PTM BIO, China), anti-TSG101 (1:500, mouse monoclonal, PTM-5108, PTM BIO, China), anti-SDCBP (1:500, rabbit monoclonal, D223488-0025, BBI LIFE SCIENCES CORPORATION, China), and anti-Calnexin as a negative control (1:1,000, rabbit monoclonal, 10427-2-AP, Proteintech). Western blotting bands were visualized by enhanced chemiluminescence (ECL) (YESEN, China).

### TEM

Mix the isolated sEVs with an equal volume of 4% paraformaldehyde (PFA) to achieve a final concentration of 2% PFA. Incubate for 30 min at room temperature. Pipette 10 µL of the fixed sEV sample onto the grid. Allow the sample to adsorb to the grid for 20 min at room temperature. Wash the grid by placing it on a drop of PBS to remove excess PFA. Place the grid on a drop of 1% glutaraldehyde for 5 min to further fix the sEVs. Wash the grid with distilled water to remove the glutaraldehyde. Stain the grid by placing it on a drop of 1% uranyl acetate for 5 min. This step provides contrast to the sEVs. Once the grid is dry, load it into the TEM instrument.

### NTA

The Vesicle suspensions with concentrations between 1 × 10^7^/mL and 1 × 10^9^/mL were examined using the Zeta View PMX 110 (Particle Metrix, Meerbusch, Germany) equipped with a 405 nm laser to determine the size and quantity of the particles we isolated. A video of 60-second duration was taken with a frame rate of 30 frames/sec, and particle movement was analyzed using NTA software (ZetaView 8.02.28).

### Transcriptomic sequencing

Plasma samples from 20 participants in the cataract and NTG groups were pooled separately. When we prepared mixed samples, 200 μL of plasma was taken from each individual sample, and a group of 20 samples were 4 mL in total. Total RNA was extracted with a Total RNA kit (Omega Bio-Tek) following the manufacturer’s instructions. The RNA fragments from plasma sEVs were detected using 2200Tape Station, to ensure that the integrity, concentration, fragment size and quality of the RNA sample meet the standards for on-machine sequencing. Next-generation RNA sequencing was performed on an Illumina HiSeq 2500 at RiboBio (Guangzhou, China). The FastQC software was used to remove adapter dimers, low complexity, and reads with lengths lower than 17 nt from the raw Illumina HiSeq 2500 sequencing to obtain high-quality clean data. The clean data were subsequently mapped with the reference genome using the HISAT2 software to obtain a map of the distribution of the whole genome reads, and the RPKM method normalized the read counts by the gene length and the total number of mapped reads, allowing for the comparison of gene expression levels between different samples. DEGseq is based on a binomial distribution and combines Fisher’s exact test with the likelihood ratio test to identify the differentially expressed genes. The BioProject Accession number is: PRJCA015141.

### Bioinformatic analyses

The volcano plot was generated by the ggplot2 R package. Kyoto Encyclopedia of Genes and Genomes (KEGG) pathway enrichment and Gene Ontology enrichment analyses were performed for differentially expressed mRNAs by the DAVID website (http://david.ncifcrf.gov/), PANTHER classification system (http://geneontology.org/), and Metascape (http://metascape.org/). We chose Fisher’s exact test to select significant pathways, and the significance threshold was defined based on its *P* value and false discovery rate (FDR). Bioinformatics prediction of target genes and functional pathway enrichment was performed by MiRpath v.3 (http://microrna.gr/miRPathv3).

### Quantitative real-time polymerase chain reaction

Total RNA was extracted by TRIzol reagent (R0016, Beyotime, China) following the manufacturer’s instructions. 2200Tape Station was employed to ensure the integrity, concentration, fragment size and quality of the RNA sample. Reverse transcription primers (stem–loop) and qPCR primers (forward and reverse) for each miRNA and mitochondrial RNA were designed based on the conserved regions of human genes, and their sequences are provided in Supplementary Tables 1 and 2. A total of 300 ng RNA was used for quantitative real-time polymerase chain reaction (qRT-PCR) reactions. HiScript III All-in-one RT SuperMix Perfect for qPCR (R333-01, Vazyme, China) was used for reverse transcription according to the manufacturer’s instructions. Reverse transcription of mitochondrial RNA was performed using the GoScript Reverse transcription System (A5004, Promega, USA). Real-time PCR was performed using Taq pro Universal SYBR qPCR Master Mix (Q712-02, Vazyme, China) and a QuantStudio 3 Real-Time PCR system (Applied Biosystems, Foster City, CA, USA). miR-30a-5p was used as an internal control to normalize the miRNA levels in EVs. The 2^ΔΔ^*^Ct^* method was used as a relative quantification strategy for validation of the relative expression of miRNA. We set a mean value of Δ*Ct* in all control samples as a standard, and then the Δ*Ct* of all samples from the control and others were deducted by the mean Δ*Ct* to obtain the individual ΔΔ*Ct*. The 2^ΔΔ^*^Ct^* method was used to calculate fold changes. We repeated each sample triplicate for qPCR validation.

### Receiver operating characteristic and correlation analyses

Logistic regression was used to establish different models as predictors of the biomarker for NTG patients. Receiver operating characteristic (ROC) analysis was utilized to assess the ability of miRNA expression to distinguish NTG patients from controls and other diseases. The optimal cutoff value was determined by maximizing the sum of sensitivity and specificity. The correlation between mean deviation (MD), visual field index (VFI), pattern standard deviation (PSD), and retinal nerve fiber layer (RNFL) with miRNA expression levels was used in the Spearman correlation analysis. Linear regression was used to characterize the relationship of the miRNA expression levels with the MD, VFI, PSD, and RNFL of the more severe eye.

### Statistical analyses

Statistical analyses were performed using statistical software (SPSS, GraphPad Prism 8.0). Data for continuous variables are presented as the means and standard deviation [mean ± standard deviation (SD)]. To evaluate the normality, we performed the Shapiro-Wilk test, which is a widely used method for testing normality in small sample sizes. Two-tailed Student’s *t* test (two groups) was used for the data analysis when the data had a normal distribution. We employed nonparametric tests such as the Mann-Whitney U test for comparing independent groups and the Wilcoxon signed-rank test for paired samples. The Mann-Whitney U test is suitable for comparing two independent datasets, while the Wilcoxon signed rank test is suitable for comparing two related conditions for the same set of samples. The nonparametric Mann–Whitney test (two groups) was used for comparison between the two groups when the data had a skewed distribution. The results are presented as the means ± standard error of mean (SEM). The *P* value was considered significant if it was less than 0.05. Supplementary Table 3 is a list of reagents and software used in this study.

## RESULTS

### Clinical characteristics and study design

**Figure 1:**
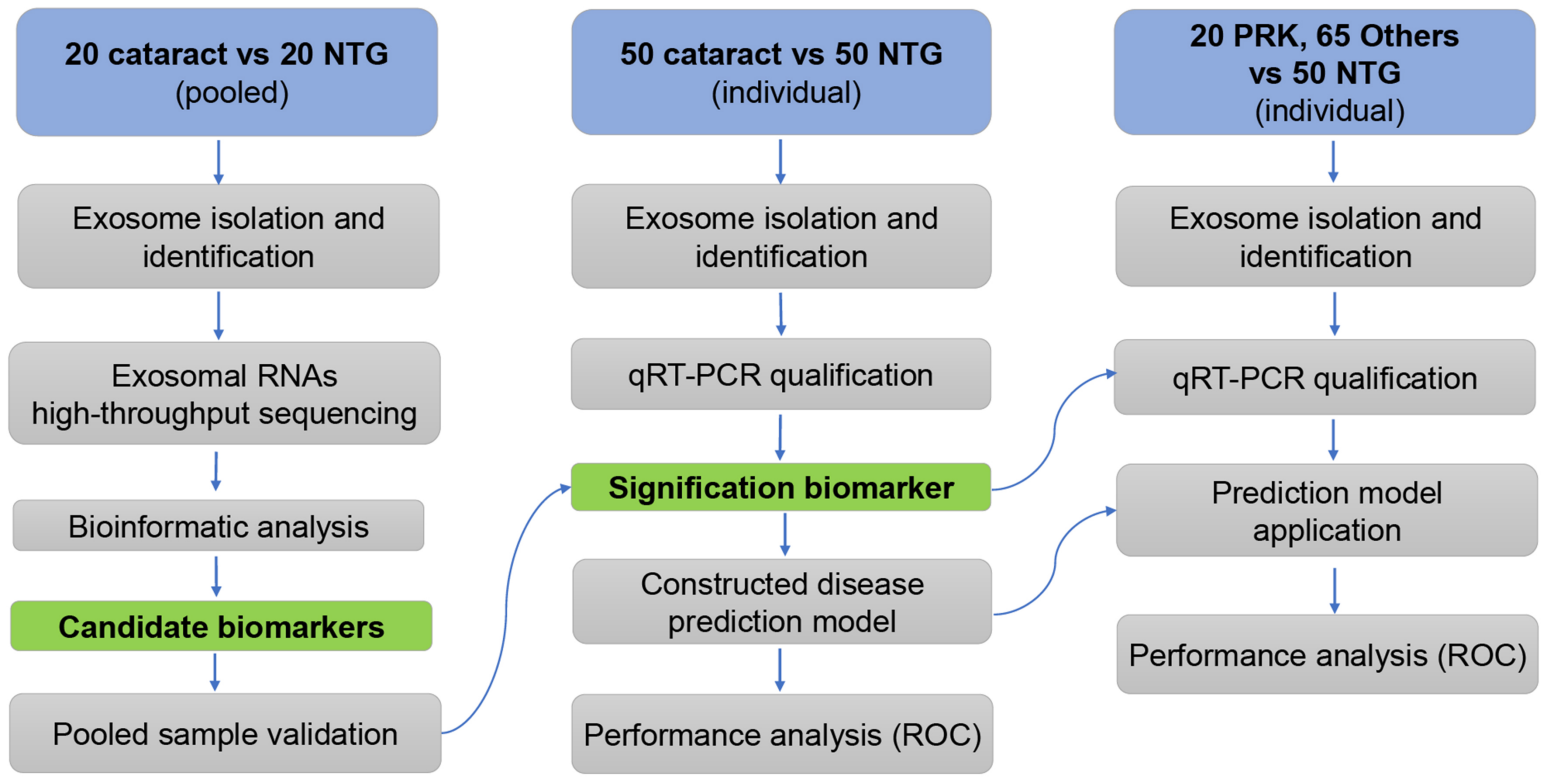
Schematic overview of the study. NTG denotes normal tension glaucoma, and cataract refers to age-related cataract patients, which were used as a control group; PRK indicates photorefractive keratectomy, representing patients with refractive errors, and “Others” refers to a control group comprising different eye diseases. NTG: Normal tension glaucoma.

The NTG patients were diagnosed by a professional physician according to the inclusion and exclusion criteria described in the methods. A total of 225 participants, including 70 NTG, 70 cataract, 20 photorefractive keratectomy (PRK), and 65 other ocular diseases [20 cases of diabetic retinopathy (DR), 15 cases of POAG, 10 cases of age-related macular degeneration (AMD), 10 cases of branch retinal venous occlusion (BRVO), and 10 cases of uveitis], were enrolled in this study. The clinical characteristics of the subjects are summarized in Table 1. A schematic representation of the study design is shown in Figure 1.

**Table 1:** The characteristics of the enrolled patients

**Characteristics**	**NTG**	**Cataracts**	***P* value**	**PRK**	***P* value**
Total (*n*)	70	70		20	
Age, mean (SD), y	49.96 (15.32)	66.06 (17.46)	< 0.0001	24.85 (6.80)	< 0.0001
Male, *n* (%)	58	58.9	0.244	85	0.023
IOP, mean (SD), mmHg	12.61 (2.67)	14.04 (3.27)	0.0033	17.58 (1.61)	< 0.0001
MD, mean (SD), dB	-7.57 (7.18)	N/A		N/A	
VFI, mean (SD)	81.38 (25.41)	N/A		N/A	
PSD, mean (SD)	5.51 (3.34)	N/A		N/A	
RNFL, mean (SD), μm	77.34 (32.86)	N/A		N/A	

NTG: Normal tension glaucoma; PRK: photorefractive keratectomy; SD: standard deviation; IOP: intraocular pressure; MD: mean deviation; N/A: not applicable; VFI: visual field index; PSD: pattern standard deviation; RNFL: retinal nerve fiber layer.

First, we used pooled plasma samples from 20 NTG patients and 20 age-related cataract patients for sEVs RNA profiling by high-throughput sequencing. We subsequently confirmed the expression levels via RT-qPCR using pooled samples to explore the most significantly altered genes that were consistent with the sequencing data. Next, individual validation of the sequencing data was conducted in 50 NTG patients and 50 cataract controls.

To further investigate the feasibility of plasma-derived sEVs miRNA as a biomarker in NTG patients, 20 PRK controls and 65 other patients with ocular diseases were enrolled to verify their specificity and sensitivity.

### sEVs mRNA signature of circulating plasma in NTG patients

**Figure 2:**
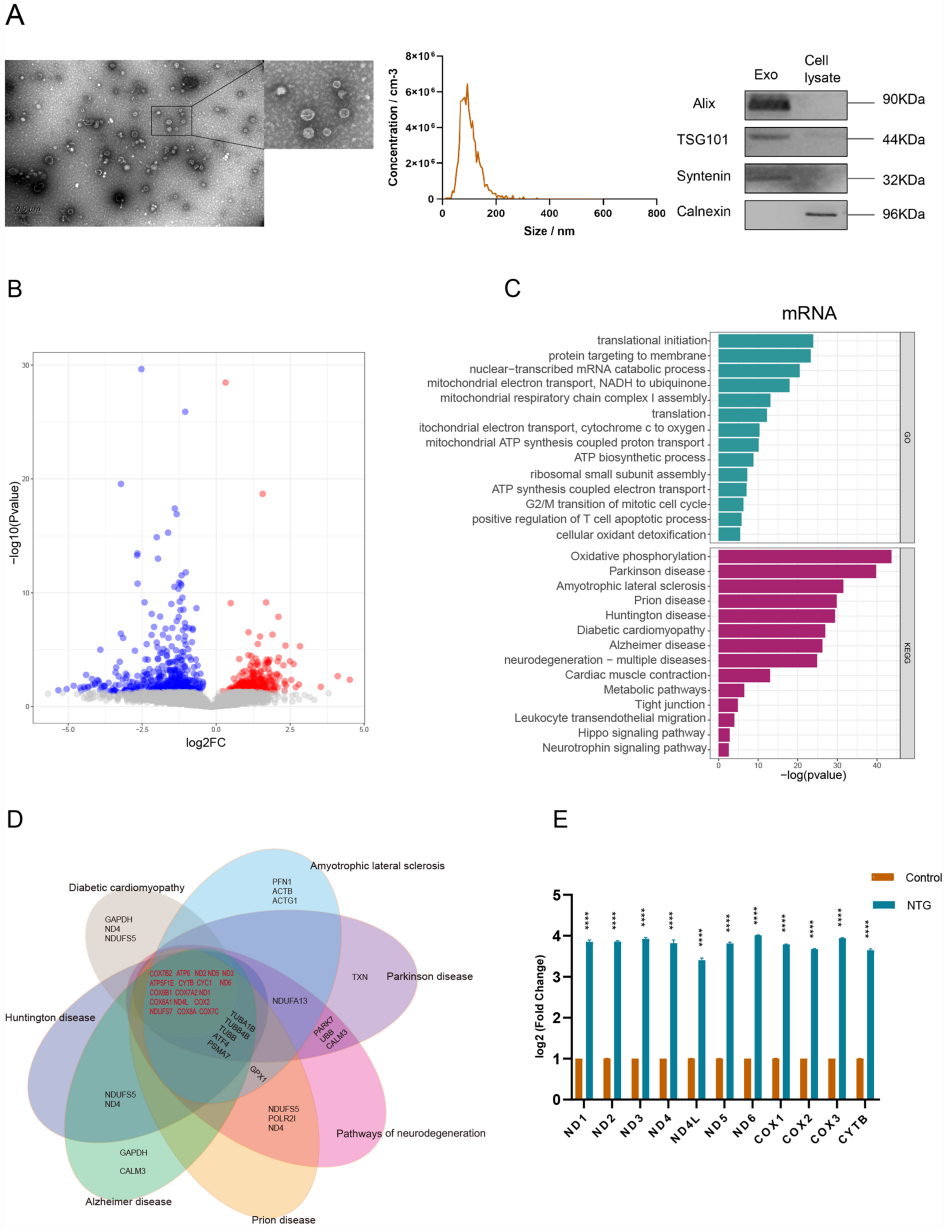
The characteristics of sEVs and the sEVs mRNA signature of plasma in NTG patients. (A) Characteristics of plasma sEVs: Transmission electron microscopy images of sEVs with scale bar = 500 nm; nanoparticle tracking analysis for a particle diameter, and immunoblotting for commonly used sEVs protein markers, which include Alix, TSG101, and syntenin; (B) Volcano plot of mRNAs in plasma sEVs. The red dots represent upregulated genes; the blue dots represent downregulated genes; (C) GO and KEGG analyses of mRNAs that were differentially expressed with a fold change ≥ 2 and *P* ≤ 0.05 between the NTG and cataract groups. The X-axis represents the -log10 (*P* value), and the Y-axis represents the top 15 functions or signaling pathways associated with each term; (D) Venn diagram of neurodegenerative disease-related pathways. Genes involved in multiple neurogenerative pathways are shown in red; (E) The expression levels of mitochondrial mRNAs in pooled plasma sEVs. The data are represented as the means ± SEM. ^****^P < 0.0001. sEVs: Small extracellular vesicles; NTG: normal tension glaucoma; GO: Gene Ontology; KEGG: Kyoto Encyclopedia of Genes and Genomes.

The plasma-derived sEVs of the participants were isolated and confirmed by multiple experiments [Figure 2A]. The results revealed a typical cup-shaped structure of sEVs under TEM and NTA with a peak size of 85.6 nm. There is significant lipoprotein contamination in the TEM samples; we will optimize our method according to MISEV standards to prevent contamination in future studies. The presence of sEVs was confirmed by the expression of the specific protein markers Alix, TSG101, and Syntenin. Calnexin is an integral membrane protein primarily located in the endoplasmic reticulum (ER) and is not expected to be present in sEVs, which are derived from endosomal compartments. Therefore, its absence in our sEVs samples serves as a critical negative control. High-throughput sequencing was conducted to discover the sEVs transcriptomes in the circulating plasma.

To understand the functional characteristics of these genes, we first focused on coding-gene mRNAs and identified 2,504 differentially expressed genes (DEGs) [Figure 2B]. Gene Ontology (GO) analysis revealed that biological processes related to mitochondrial function were significantly enriched [Figure 2C]. Moreover, KEGG pathway analysis also revealed that multiple common neurodegenerative disease-related pathways were enriched dramatically in plasma-derived sEVs of NTG patients [Figure 2C]. The Venn diagram reveals that the mitochondrial-encoding genes were involved in common neurodegenerative diseases [Figure 2D]. We then confirmed the expression levels of sEVs mitochondrial-encoding genes in the pooled plasma samples [Figure 2E]. We also used Integrative Genomics Viewer (IGV) software and PolyPhen-2 software to analyze the mutation sites of mitochondrial fragments of mRNA and predict the probable damage to these single nucleotide polymorphisms (SNPs) [Supplementary Figure 1]. We found two mutations with high pathogenicity.

Accumulating evidence has shown that mitochondrial dysfunction is closely related to neurodegeneration. NTG is also a known neurodegenerative disease, and its pathogenesis is closely related to mitochondrial dysfunction. Interestingly, our novel study revealed that mitochondrial function-related biological processes and pathways related to common neurodegenerative diseases were present in the plasma-derived sEVs of NTG patients, reinforcing the importance of circulating plasma sEVs detection in clinical diagnosis and disease features.

### sEVs miRNA screening of circulating plasma in NTG patients

**Figure 3:**
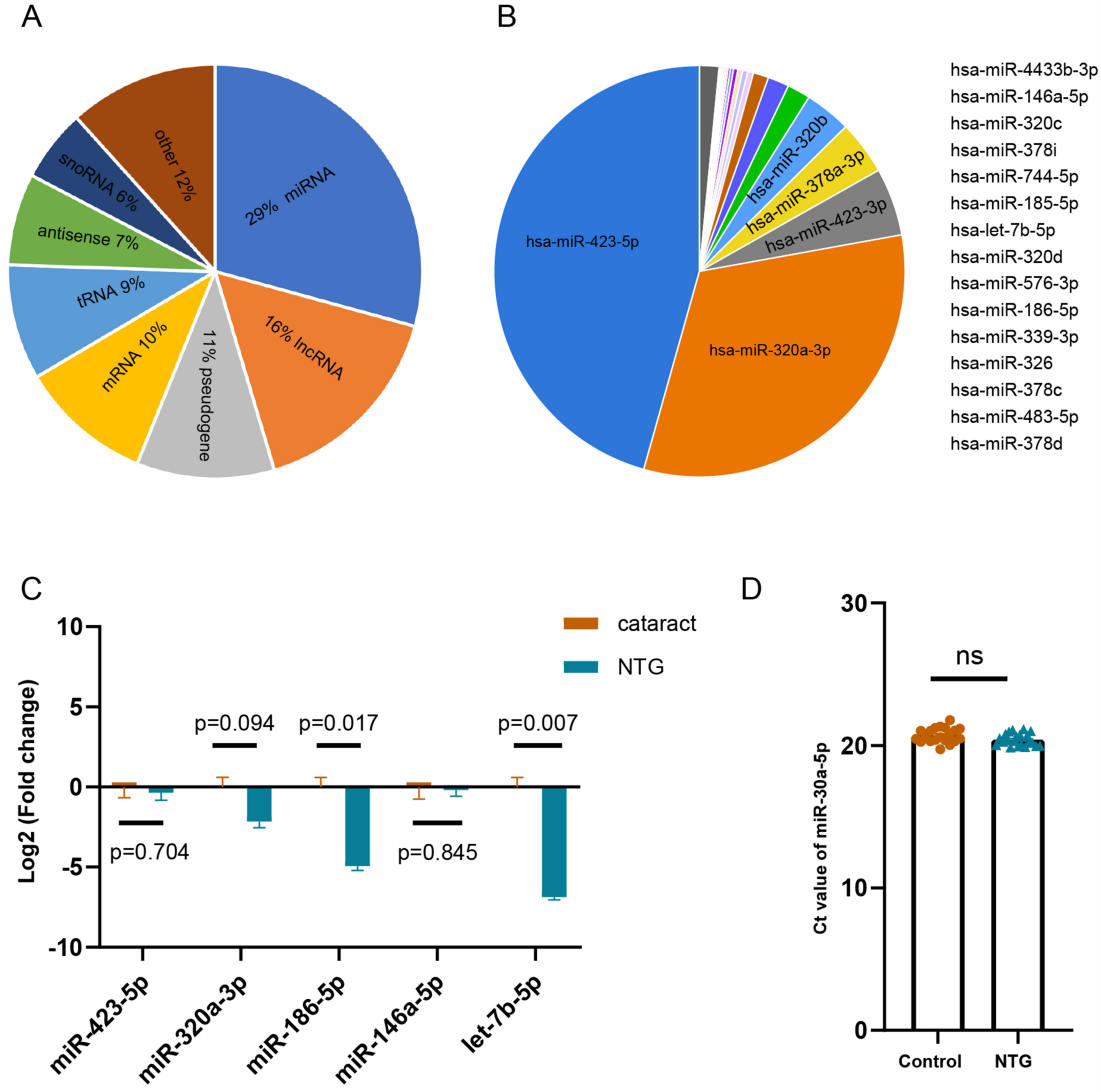
sEVs miRNA screening of plasma from NTG patients. (A) Distribution of differentially expressed RNAs identified in plasma sEVs from NTG patients compared with those from cataract patients; (B) Percentages of sEVs miRNA in plasma from NTG patients; (C) Relative expression of candidate miRNAs in the pooled samples. Data are represented as the mean ± SEM; (D) *Ct* values of miR-30a-5p in the control and NTG groups. The data are represented as the means ± SEM. sEVs: Small extracellular vesicles; NTG: normal tension glaucoma; SEM: standard error of mean.

According to the plasma-derived sEVs RNA profile, we showed that miRNA was most abundant, followed by lncRNA, pseudogene, and mRNA [Figure 3A]. To further explore the contribution of miRNAs, we found that the top 20 differentially expressed miRNAs accounted for 98% of all miRNA expression [Figure 3B]. We also conducted biology pathway analyses for the top 20 miRNAs, and selected those associated with neurodegenerative diseases pathways, including miR-423-5p, miR-146a-5p, miR-320a-3p, miR-186-5p, and let-7b-5p; we speculated that these miRNAs are potential biomarkers for NTG, and can also reflect the pathology. On the basis of the abundance of miRNAs in plasma-derived sEVs, it is more reasonable to detect miRNAs in trace samples. For this reason, we conducted a quantitative analysis using pooled plasma samples to screen candidate sEVs miRNAs and found that let-7b-5p levels were significantly lower in NTG patients than in controls, which was also consistent with the sequencing data [Figure 3C]. Non-differentially expressed miRNAs, including miR-423-5p and miR-146a-5p, were excluded; miR-320a-3p was excluded because its expression trend was inconsistent with sequencing; and miR-186-5p was excluded because its *Ct* value was more than 30, indicating low expression in the samples. According to the results of previous studies, miR-30a-5p acts as a stable sEVs internal reference gene^[33,34]^. The levels of miR-30a-5p in plasma-derived sEVs were relatively stable between the groups [Figure 3D], so we used an internal reference method for subsequent individual validation.

**Figure 4:**
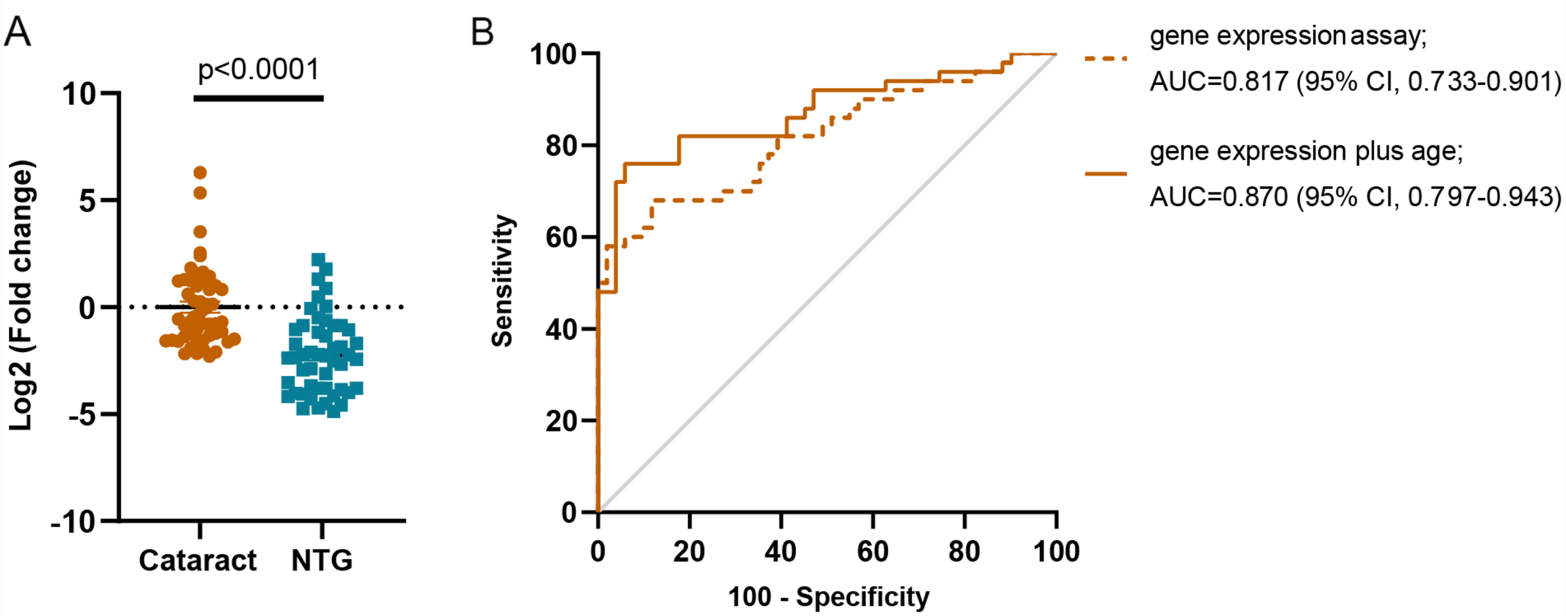
Let-7b-5p was used in the individual validation set, and prediction models were constructed. (A) The relative expression of let-7b-5p in individual validation set, which included 50 cataract patients and 50 NTG patients. The data are represented as the mean ± SEM; (B) ROC curves were generated for individual validation sets with different prediction models. NTG: Normal tension glaucoma; SEM: standard error of mean; ROC: receiver operating characteristic.

To further confirm the let-7b-5p levels in plasma-derived sEVs, we recruited 50 NTG and 50 cataract controls and performed qRT-PCR analysis at the individual level. In the results, we found that the expression levels of sEVs let-7b-5p were significantly downregulated in the NTG patients, which was consistent with the results in pooled samples [Figure 4A]. To investigate the possibility of let-7b-5p levels as a biomarker, we used patients’ gender, age, and expression levels of let-7b-5p as influencing factors to construct logistic regression models. The factors of gender and age (age < 40 years or age ≥ 40 years) are binary variables, and let-7b-5p levels were continuous variables. A logistic regression model revealed that age can be a main risk factor (OR: 8.185, 95%CI: 2.164-30.959, *P* = 0.002), but let-7b-5p levels can be a protection factor (OR: 0.425, 95%CI: 0.296-0.612, *P* = 0.0001). We then performed ROC curve analysis to evaluate diagnostic ability. ROC curve analysis revealed that the area under the curve (AUC) was 0.817 (95%CI: 0.733-0.901, *P* < 0.0001*)* with 98% sensitivity and 58% specificity and the AUC combined with age was 0.870 (95%CI: 0.797-0.943, *P* < 0.0001) with 76% sensitivity and 94.1% specificity [Figure 4B]. This result indicated that the performance of the prediction model with age as a factor improved the accuracy of NTG diagnosis.

### Let-7b-5p can discriminate NTG patients from other patients with ophthalmic disease

**Figure 5:**
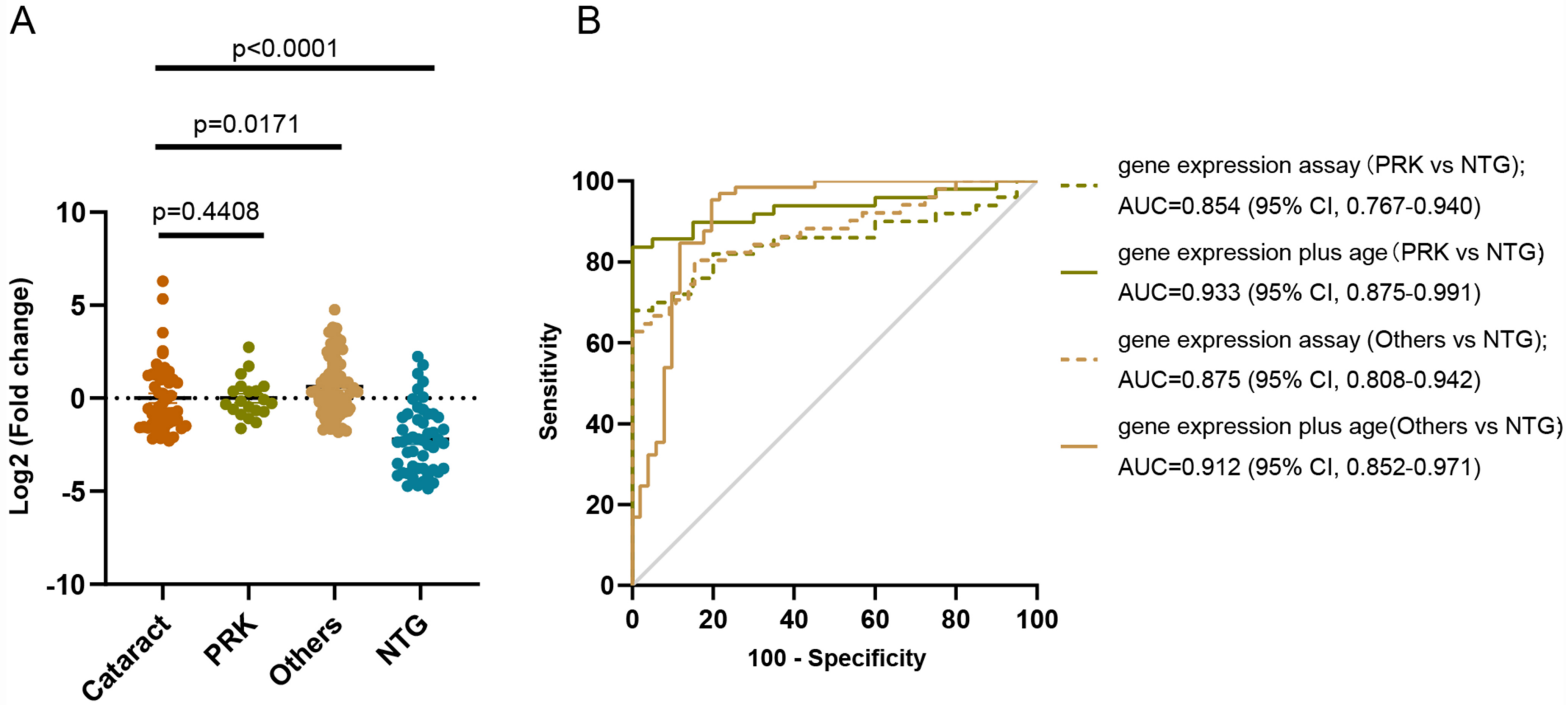
Let-7b-5p can discriminate NTG patients from patients with other ophthalmic diseases. (A) The relative expression of let-7b-5p in individual samples from 50 cataract patients, 20 PRK patients, 65 patients with other ophthalmic diseases, and 50 NTG patients. Data are represented as the mean ± SEM; (B) The ROC curve was generated using different prediction model parameter applications. NTG: Normal tension glaucoma; PRK: photorefractive keratectomy; SEM: standard error of mean; ROC: receiver operating characteristic.

To further verify the diagnostic efficiency and clinical application value, we recruited patients in both the PRK group and the group with other ophthalmic diseases. Considering that the mean age of the cataract patients was older than that of the NTG patients and that age is an influencing factor for disease diagnosis, we enrolled PRK patients as younger healthy controls to evaluate the diagnostic value. As expected, the quantitative analysis demonstrated that sEVs let-7b-5p levels were markedly lower in the circulating plasma of NTG patients than in that of both the PRK group and the other disease groups [Figure 5A].

To further verify the specificity of the biomarker, we investigated other eye diseases, including POAG, DR, AMD, RVO, and uveitis. This cohort included a total of 65 patients who were diagnosed by an experienced doctor from the eye hospital affiliated with Wenzhou Medical University. Consolidation of three different control groups of individual validation demonstrated that the expression levels of plasma-derived sEVs let-7b-5p were significantly reduced in NTG patients (*n* = 50) than in controls, including 50 patients with cataracts, 20 patients with PRK, and 65 patients with other disease [Figure 5A]. ROC analysis revealed that the AUC between PRK and NTG was 0.854 (95%CI: 0.767-0.940, *P* < 0.0001) with 100% sensitivity and 67.3% specificity and 0.933 (95%CI: 0.875-0.991, *P* < 0.0001) with 83.7% sensitivity and 100% specificity, respectively, when combined with age. Meanwhile, the AUC between others and NTG was 0.875 (95%CI: 0.808-0.942, *P* < 0.0001) with 84.6% sensitivity and 80.4% specificity and 0.912 (95%CI: 0.852-0.971, *P* < 0.0001) with 80.4% sensitivity and 95.4% specificity, respectively [Figure 5B]. These results suggest that plasma-derived sEVs let-7b-5p levels can be a promising biomarker for NTG diagnosis and further confirm the specificity of plasma-derived sEVs let-7b-5p levels in NTG diagnosis, but a large number of samples and multicenter studies are needed in the future.

### Let-7b-5p expression levels reflect NTG clinical features and severity

To evaluate the feasibility of the clinical application of plasma-derived sEVs let-7b-5p levels as a biomarker in NTG diagnosis, we stratified NTG patients into two groups (28 low-level and 22 high-level), using the mean expression levels as the cutoff value. Table 2 shows the characteristics of the patients and their clinical indices. We selected the clinical indicators of patients to analyze their correlation with let-7b-5p expression levels in the NTG group. It has been demonstrated that let-7b-5p levels have a significant linear trend with these clinical indicators, including visual field defects (MD, PSD, and VFI) and RNFL thickness [Supplementary Figure 2A-D]. Because this dataset had a skewed distribution, we chose Spearman’s test to calculate the correlation coefficient, and the results showed that the expression levels of let-7b-5p were significantly correlated with these clinical indicators [Supplementary Figure 2E].

**Table 2:** Relation between let-7b-5p expression levels and the clinicopathologic characteristic of NTG patients

**Variable**	**Exosomal miRNA grade (log2FC)**		***P* value**
	**Low (*n* = 28)**	**High (*n* = 22)**	
Age, years (SD)	54.96 (14.01)	42.50 (14.11)	0.0031
Sex (M:F)	15:13	14:8	0.474
MD (SD)	-9.323 (7.849)	-5.876 (6.536)	0.0106
PSD (SD)	6.311 (3.178)	4.534 (3.351)	0.0147
VFI (SD)	77.91 (25.76)	85.80 (24.84)	0.0052
RNFL (SD)	65.59 (26.57)	92.45 (34.54)	0.0056

NTG: Normal tension glaucoma; SD: standard deviation; MD: mean deviation; PSD: pattern standard deviation; VFI: visual field index; RNFL: retinal nerve fiber layer.

**Figure 6:**
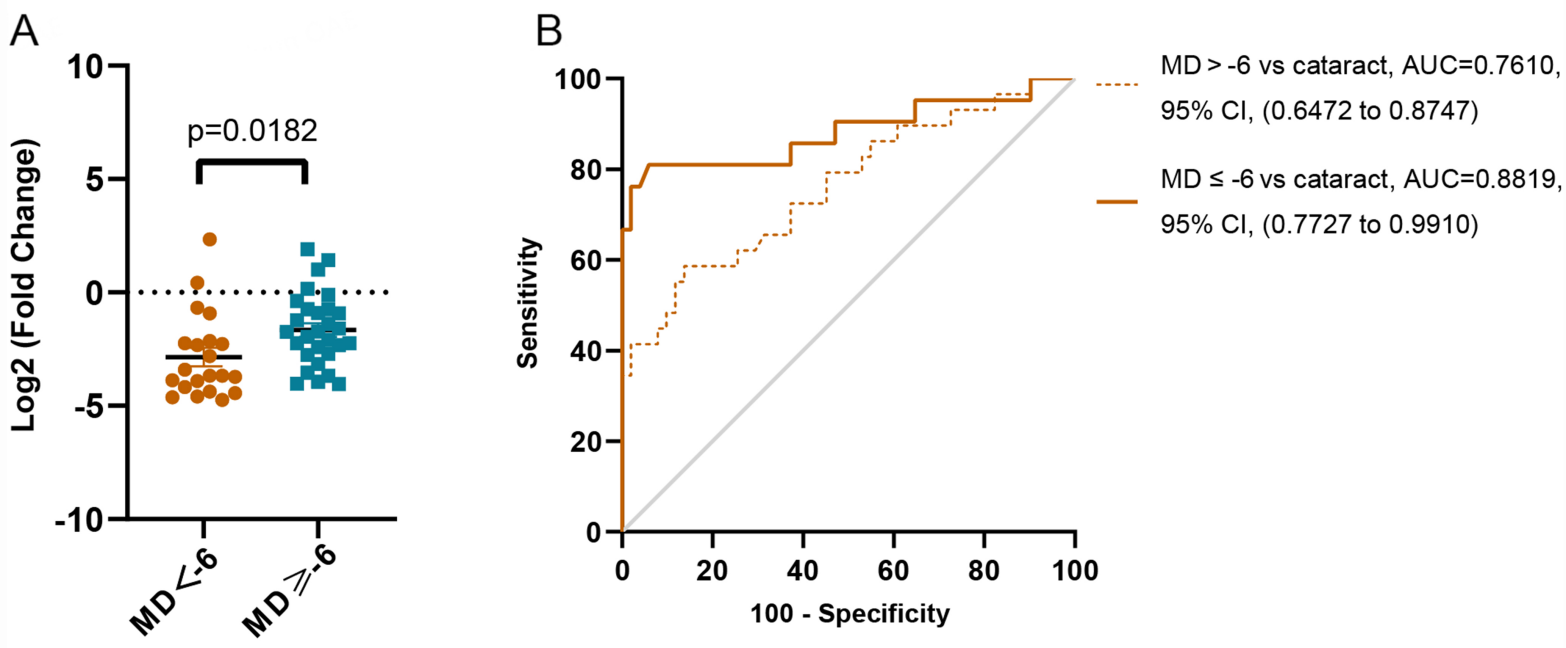
The plasma sEVs let-7b-5p level is a significant diagnostic factor in severe NTG patients. (A) According to the MD value, we stratified the NTG patients into two groups. The relative expression levels of let-7b-5p in the two groups. Data are represented as the mean ± SEM; (B) ROC curve analysis was performed to analyze the ability of let-7b-5p in terms of evaluating the severity of NTG. sEVs: Small extracellular vesicles; NTG: normal tension glaucoma; MD: mean deviation; SEM: standard error of mean; ROC: receiver operating characteristic.

The NTG patients were further divided into mild (MD > -6 dB) and moderate to severe (MD ≤ -6 dB) groups according to the MD value of the visual field test. As shown in Figure 6A, the expression levels of let-7b-5p were significantly different between the two groups. The ROC curve analysis also found an AUC value of 0.761 (95%CI: 0.647-0.874) with 86.3% sensitivity and 58.6% specificity between mild NTG patients and those with cataracts, and it found an AUC of 0.882 (95%CI: 0.772-0.991), with 94.1% sensitivity and 81% specificity between the moderate to severe NTG patients and those with cataracts [Figure 6B]. These results suggest that there is a significant correlation between plasma-derived sEVs let-7b-5p levels and clinical indicators, and that let-7b-5p can be useful for monitoring disease progression.

## DISCUSSION

Glaucoma is a major leading cause of blindness worldwide^[35]^. NTG is a multifactorial disease that is characterized by progressive RGC degeneration, leading to irreversible visual field damage with a normal range of IOP^[36]^. An increasing amount of evidence suggests a relationship between glaucoma and degenerative diseases of the central nervous system^[11,12,37-39]^. Severe glaucomatous visual field defects are associated with Alzheimer’s disease (AD) and Parkinson’s disease (PD)^[40-42]^. Moreover, studies have also reported that systemic immune regulation is altered during glaucoma progression^[4]^. Thus, glaucoma may be not only a local ocular disease but also a systemic regulatory mechanism involved in disease progression.

One of the purposes of this study was to explore the characteristics of circulating plasma sEVs and to explain the mechanism underlying the systemic regulation of NTG. Interestingly, several neurodegenerative disease-related pathways, especially those related to the biological process of mitochondrial dysfunction, were enriched in the circulating plasma sEVs of NTG patients [Figure 2C and D]. We further confirmed that the expression levels of mitochondrial coding genes were upregulated in circulating sEVs of NTG patients [Figure 2E].

Interestingly, we also detected a high frequency of SNPs in the mitochondrial coding genes of NTG patients. In this study, we used the IGV software to analyze the sequencing data of pooled samples from 20 patients in each group, and we subsequently used the PolyPhen-2 software to predict the possibility of these SNPs [Supplementary Figure 1], among which 2 mutations with high pathogenicity were found: m. C5178A (Leu- > Met) and m. C5306T (Pro- > Leu). We speculated that these SNPs may contribute to NTG development by mediating mitochondrial dysfunction, but further studies are needed to prove this hypothesis in the future.

Numerous previous studies have shown that mitochondrial dysfunction is involved in neurodegenerative diseases of the central nervous system^[43-46]^. Mitochondrial dysfunction triggers programmed axon death^[13]^; mitochondrial DNA mutation is related to tumorigenesis via mitochondrial dysfunction^[47]^. A previous study also demonstrated that mitochondrial proteins were enriched in the plasma sEVs of patients^[48]^. Combining this evidence with our findings, we propose that the development of NTG is correlated with mitochondrial dysfunction and that the disease characteristics can be detected in circulating plasma sEVs.

Integrating findings from recent studies on lncRNA regulation, exosomal biomarkers, and peripheral nerve regeneration offers deeper insights into the potential of exosomal let-7b-5p as a biomarker for NTG. Mancheng *et al.* emphasized the critical role of lncRNAs in metastasis through processes such as epithelial-mesenchymal transition (EMT)^[49]^. Similarly, the regulation of sEVs miRNAs, like let-7b-5p, could influence cellular mechanisms in NTG, potentially affecting disease progression. Furthermore, the therapeutic potential of exosomes is highlighted by their ability to enhance nerve regeneration^[50]^, underscoring the broader applicability of sEVs RNA in clinical settings and suggesting that sEVs let-7b-5p might also have therapeutic implications for NTG by reflecting mitochondrial dysfunction. Additionally, the utility of exosome profiling in the early diagnosis and treatment of cancer supports our findings that sEVs let-7b-5p levels correlate with NTG progression, endorsing its potential as a noninvasive diagnostic biomarker^[51]^.

In fact, up to half of RGCs degenerate when diagnosed clinically^[14]^, reinforcing that early diagnosis is urgent. Unfortunately, there are no useful noninvasive body fluid-based biomarkers for the early diagnosis and prognosis of ocular diseases thus far. Another purpose of this study was to identify a noninvasive and easy-to-use clinical biomarker that can be used for early diagnosis and large-scale population screening.

Therefore, we explored a noninvasive biopsy biomarker based on plasma sEVs and identified the miRNA let-7b-5p, which can be used as a biomarker for NTG diagnosis or monitoring disease progression [Figures 4-6]. Most studies have focused on miRNAs because they are enriched in sEVs and may also be easy to detect^[52,53]^. Our study also demonstrated that miRNAs were enriched in the plasma sEVs of NTG patients [Figure 3]. Among the differentially expressed miRNAs identified via high-throughput small RNA sequencing of the plasma sEVs, we identified and verified that the expression levels of let-7b-5p were downregulated in NTG patients, which is consistent with the sequencing results [Figures 4 and 5].

To consider the influence of age on the relationship between NTG and cataracts, we further compared patients with PRK as the young controls with patients with age-related cataracts who were older than the NTG patients. The results revealed that there was no difference in the expression levels of let-7b-5p between cataract patients and patients with PRK, but let-7b-5p expression was significantly decreased in patients with NTG [Figure 5]. To further verify the specificity of this method in NTG patients, we also determined that the plasma sEVs let-7b-5p levels were slightly higher in patients with other ocular diseases than in those with cataracts [Figure 5]. In addition, we found that decreased levels of let-7b-5p were correlated with visual field defects [Figure 6 and Table 2], suggesting that it can be useful for monitoring disease progression in NTG patients. Overall, combining the expression levels of mitochondrial coding genes and let-7b-5p in circulating plasma sEVs can be a reliable and promising noninvasive biomarker for NTG diagnosis and prognosis.

Limitations of this study include the lack of healthy controls because of difficulties in ethics and informed consent, and multicenter and prospective research is needed for future studies. In addition, we considered that proteomic analysis would provide more information about the sEVs signature, probably favoring the progression of disease pathology. From a clinical application perspective, it is also necessary to establish standardized operating procedures and technical training for the extraction, identification, and quantitative analysis of EVs.

The molecules released by diseased eye cells are present at extremely low levels in the circulating blood, so it is difficult to detect their signals and accurately quantify them. For this reason, noninvasive biopsy-based biomarkers for ocular diseases are difficult for researchers and clinicians to recognize. Nevertheless, researchers have explored the potential of peripheral blood-based biomarkers^[22]^. Our previous studies have also reported plasma-based biomarkers for ocular diseases such as glaucoma and uveitis^[54,55]^. Research suggests that POAG and NTG accompany systemic symptoms such as the central neurodegenerative diseases AD and PD^[10,12,41,56]^. Therefore, the molecules found in circulating blood may come not only from ocular cells but also from the central nervous system.

On the other hand, sEVs have more advantages in noninvasive body fluid biomarker applications; for example, the detected molecules not only originate from diseased ocular cells but are also products after reactions through paracrine and endocrine mechanisms^[57]^. Moreover, sEVs contain molecules (RNA, DNA, and protein) that are more stable than free molecules in blood because the bilayer lipid membrane can prevent their degradation^[58,59]^. In this study, we pooled 200 μL plasma of each patient for high-throughput sequencing, and then validated the expression levels of candidate biomarkers at the individual level. We believe that the application of sEVs biomarkers for noninvasive and efficient detection of disease has good prospects.

In summary, our novel study of circulating plasma sEVs provides new insight into neurodegenerative disease research on glaucomatous optic neuropathy. Profiling circulating plasma sEVs when fresh pathological retina or optic nerve tissues cannot be obtained is helpful for understanding the characteristics or systemic regulation of fundus diseases, and detection of sEVs mitochondrial coding genes and let-7b-5p in circulating plasma is a useful strategy for noninvasive early diagnosis or the monitoring of disease progression in NTG patients.

## DECLARATIONS

### Availability of data and materials

The data presented in this study are openly available in BioProject, the BioProject Accession number: PRJCA015141.

### Copyright

© The Author(s) 2024.
