# Supplementary material for: Transcriptomic analysis of plasma-derived small extracellular vesicles reveals the pathological characteristics of normal tension glaucoma
Source: Extracell Vesicles Circ Nucl Acids. 2024 Aug 19;5(3):438–54. doi: 10.20517/evcna.2024.45 (PMC11648459; doi:10.20517/evcna.2024.45)
Supplement: Supplementary file 1 [file evcna-5-3-438-SupplementaryMaterials.pdf]

Supplementary Materials

Transcriptomic analysis of plasma-derived small extracellular vesicles reveals the pathological characteristics of normal tension glaucoma

Sheng-Lan Xu[1,#], Jun-Hua Li[1,#], Wen-Meng Zhang[1], Meng-Jun Fu[1], Hui-Min Xing[1], Hua Ma[1], Xian-Hui Gong[2], Rong-Han Wu[2], Yuan-Bo Liang[2], Ren-Zhe Cui[3], Zai-Long Chi[1,2]

[1]State Key Laboratory of Ophthalmology, Optometry and Visual Science, Eye Hospital, Wenzhou Medical University, Wenzhou 325027, Zhejiang, China.
[2]National Clinical Research Center for Ocular Diseases, Eye Hospital, Wenzhou Medical University, Wenzhou 325027, Zhejiang, China.
[3]Department of Ophthalmology, Affiliated Hospital of Yanbian University, Yanji 133001, Jilin, China.
[#]Authors contributed equally.

Correspondence to: Dr. Zai-Long Chi, State Key Laboratory of Ophthalmology, Optometry and Visual Science, Eye Hospital, Wenzhou Medical University, 270 West Xueyuan Road, Wenzhou 325027, Zhejiang, China. Email: zailong.chi@eye.ac.cn

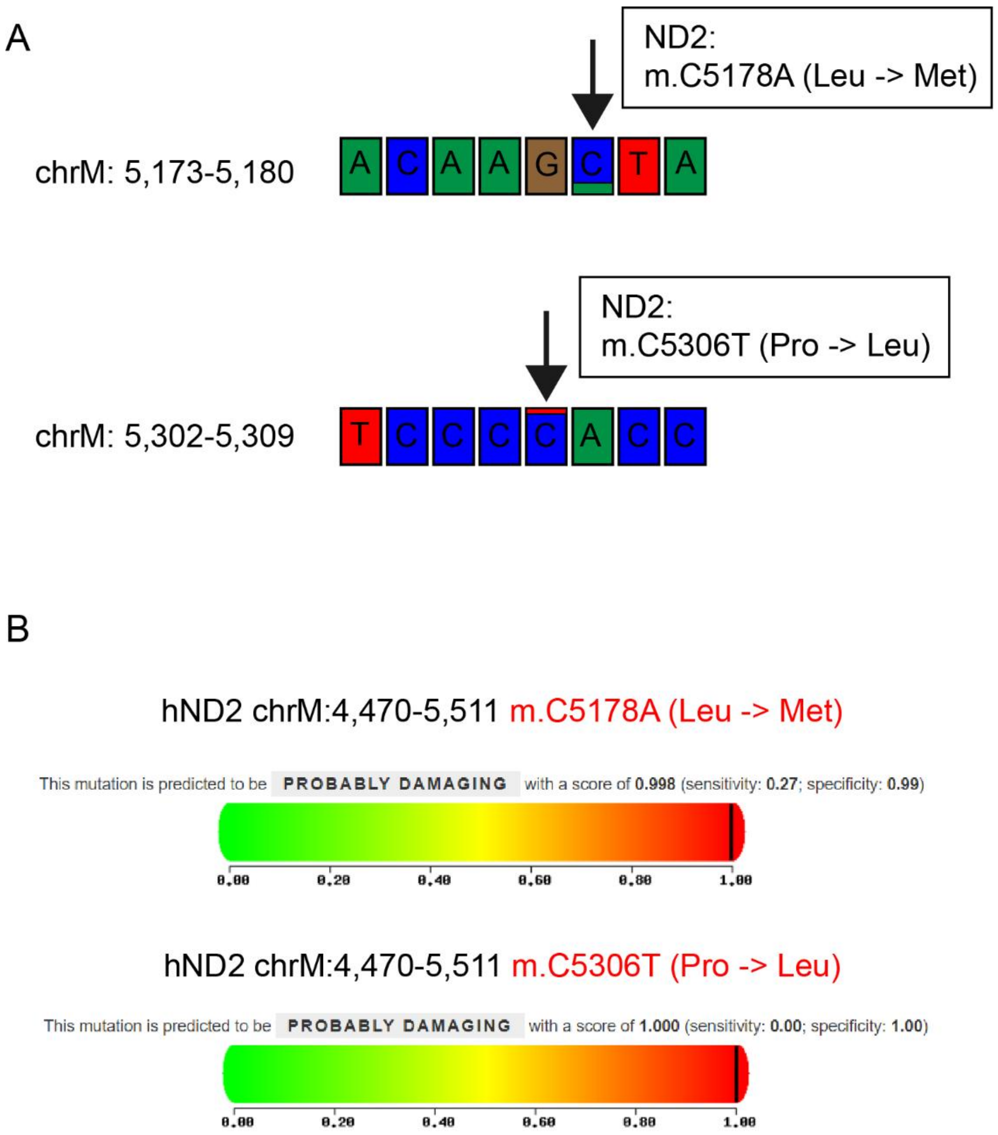

Supplementary Figure 1. Predictive analysis of pathogenicity of mutant sites of mitochondrial fragments of mRNA. (A) Integrative Genomics Viewer (IGV) software analyzed the frequency of mutation sites of mitochondrial fragments of mRNA. The black arrows indicate the mutation sites; (B) PolyPhen-2 software predicted the probably damaging of these SNPs with a score.

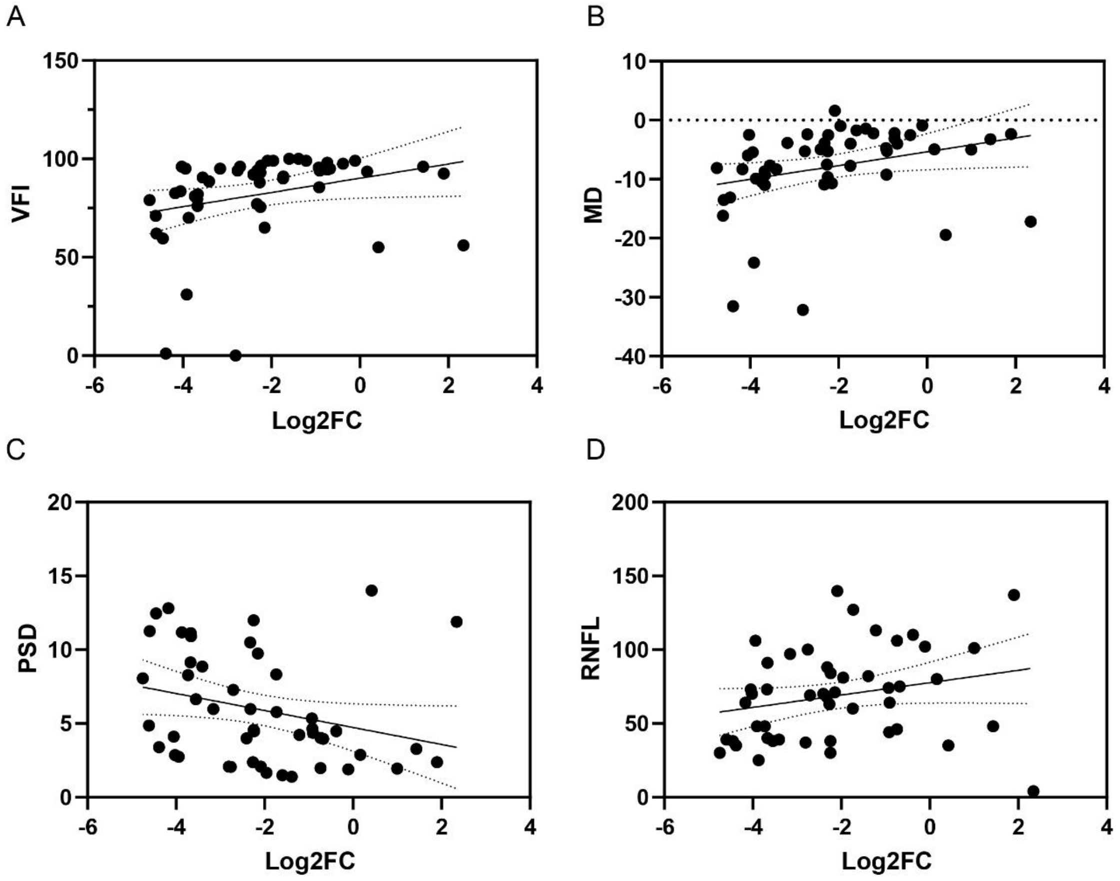

E

Clinical indicator	Spearman coefficient	*P* value
Hsa-let-7b-5p (log2 transformed)		
VFI	0.465	0.001
MD	0.479	0.0001
PSD	-0.358	0.012
RNFL	0.325	0.024

Supplementary Figure 2. The correlation between pathological characteristics and expression levels of let-7b-5p. (A-D) The linear trend between NTG clinical features and expression levels of let-7b-5p; (E) The spearman coefficient indicated the correlation between pathological characteristics and expression levels of let-7b-5p.

Supplementary Table 1. The sequence of sEVs miRNAs

Primer	Primer sequence (5' - 3')
hsa-miR-423-5p-R	CTCAACTGGTGTCGTGGAGTCGGCAATTCAGTTGAGAAAG
hsa-miR-423-5p-F	ACACTCCAGCTGGGTGAGGGGCAGAGAGCGA
hsa-miR-423-5p-R	TGGTGTCGTGGAGTCG
hsa-miR-320a-3p-R	CTCAACTGGTGTCGTGGAGTCGGCAATTCAGTTGAGTCGC
hsa-miR-320a-3p-F	ACACTCCAGCTGGGAAAAGCTGGGTTGAGA
hsa-miR-320a-3p-	TGGTGTCGTGGAGTCG
hsa-miR-186-5p-R	CTCAACTGGTGTCGTGGAGTCGGCAATTCAGTTGAGAGCC
hsa-miR-186-5p-F	ACACTCCAGCTGGGCAAAGAATTCTCCTTT
hsa-miR-186-5p-R	TGGTGTCGTGGAGTCG
hsa-let-7b-5p-RT	CTCAACTGGTGTCGTGGAGTCGGCAATTCAGTTGAGAACC
hsa-let-7b-5p-F	ACACTCCAGCTGGGTGAGGTAGTAGGTTGT
hsa-let-7b-5p-R	TGGTGTCGTGGAGTCG
hsa-miR-30a-5p-R	CTCAACTGGTGTCGTGGAGTCGGCAATTCAGTTGAGCTTCC
hsa-miR-30a-5p-F	ACACTCCAGCTGGGTGTAAACATCCTCGAC
hsa-miR-30a-5p-R	TGGTGTCGTGGAGTCG

Supplementary Table 2. The sequence of mitochondrial fragments of mRNA

Primer	Forward primer (5'-3')	Reverse primer (5'-3')
MT-ND1	GCCTCCTATTTATTCTAGCC	AGCCACTTATTAGTAATGTTG
MT-ND2	GGCCTAGAAATAAACATGC	TGCCTTGGGTAACCTC
MT-ND3	TACCACAACTCAACGGCTA	GATGACATAACTATTAGTGGC
MT-ND4	CGCAGTACTCTTAAAACTAGGC	CAATGAGCGATTTTAGGTCT
MT-ND4L	CCTCTTAGCCAATATTGTGC	TATGTACGTAGTCTAGGCCAT
MT-ND5	TTCTTACTCATCCGCTTCCAC	AGCACATAAATAGTATGGCTT
MT-ND6	CTCCCGAATCAACCCT	GATATACTACAGCGATGGCTA
MT-COX1	CCCCATTCTATACCAACACC	TGCTCACACGATAAACCC
MT-COX2	CCCCATTATTCCTAGAACCAG	TTTCAGAGCATTGACCGTA
MT-COX3	AGTCTAATAGAAAACAACCGAA	CCAATAATGACGTGAAGTCC
MT-CYTB	TCTCCGCATGATGAAACTTCG	AGAAATGATCCGTAATATAGGC
MT-ATP6	TGATTATAGGCTTTCGCTCT	GCTTCCAATTAGGTGCAT

Supplementary Table 3. List of reagents and software used in this study

REAGENT or RESOURCE	SOURCE	IDENTIFIER
**Biological samples**		
Blood samples for analysis were collected from 225 patients recruited in the trail	This manuscript	N/A
**Regents**		
Exosome Isolation Reagent	RiboBio, GuangZhou, China	https://www.ribobio.com/product_detail/?sku=C10110-2
anti-Alix antibody	PTM BIO, HangZhou, China	http://www.ptm-biolab.com.cn/productDetail.html?id=7084
anti-Tsg101	PTM BIO, HangZhou, China	http://www.ptm-biolab.com.cn/productDetail.html?id=4806
anti-Syntenin	BBI, China	https://www.sangon.com/productDetail?productInfo.code=D623488
anti-Calnexin	proteintech	https://www.ptgcn.com/products/CANX-Antibody-10427-2-AP.htm
Total RNA kit	Omega	
Trizol reagent	Beyotime, ShangHai, China	https://www.beyotime.com/product/R0016.htm
HiScript III All-in-one RT SuperMix	Vazyme, China	https://www.vazyme.com/product/326.html
GoScript Reverse transcription System	Promega, USA	https://wechat.promega.com.cn/content/details61_31904.html
Taq pro Universal SYBR qPCR Master Mix	Vazyme, China	https://www.vazyme.com/product/335.html
**Software and algorithms**		
R studio		https://cloud.r-project.org/
Integrative genomics viewer software (IGV)	Robinson et al., 2011[1]	https://igv.org/
Ployphen-2 software	Adzhubei et al., 2010[2]	http://genetics.bwh.harvard.edu/pph2/
DAVID website	DAVID Bioinformatics Team	http://david.ncifcrf.gov/
PANTHER	Mi et al., 2019[3]	http://geneontology.org/
Metascape	Zhou et al., 2019[4]	http://metascape.org/
MiRpath v.3	Vlachos et al., 2015[5]	http://microrna.gr/miRPathv3
IBM SPSS Statistics 20		https://www.ibm.com/support/pages/downloading-ibm-spss-statistics-20
Prism version 8.0.2		https://www.graphpad.com/updates/prism-802-release-notes
